# BPIFB1 (LPLUNC1) inhibits radioresistance in nasopharyngeal carcinoma by inhibiting VTN expression

**DOI:** 10.1038/s41419-018-0409-0

**Published:** 2018-03-22

**Authors:** Fang Wei, Le Tang, Yi He, Yingfen Wu, Lei Shi, Fang Xiong, Zhaojian Gong, Can Guo, Xiayu Li, Qianjin Liao, Wenling Zhang, Qianxi Ni, Jia Luo, Xiaoling Li, Yong Li, Cong Peng, Xiang Chen, Guiyuan Li, Wei Xiong, Zhaoyang Zeng

**Affiliations:** 1 The Key Laboratory of Carcinogenesis of the Chinese Ministry of Health, Xiangya Hospital, Central South University, Changsha, Hunan China; 2 The Key Laboratory of Carcinogenesis and Cancer Invasion of the Chinese Ministry of Education, Cancer Research Institute, Central South University, Changsha, Hunan China; 3 Hunan Key Laboratory of Nonresolving Inflammation and Cancer, Disease Genome Research Center, The Third Xiangya Hospital, Central South University, Changsha, Hunan China; 4 Hunan Key Laboratory of Translational Radiation Oncology, Hunan Cancer Hospital and the Affiliated Cancer Hospital of Xiangya School of Medicine, Central South University, Changsha, Hunan China; 5 Department of Cancer Biology, Lerner Research Institute, Cleveland Clinic, Cleveland, OH USA

## Abstract

Bactericidal/permeability-increasing-fold-containing family B member 1 (BPIFB1, previously named LPLUNC1) is highly expressed in the nasopharynx and significantly downregulated in nasopharyngeal carcinoma (NPC). Low expression is also associated with poor prognosis in patients with NPC. Radiotherapy is a routine treatment for NPC; however, radioresistance is a major cause of treatment failure. Thus, we aimed to investigate the role of BPIFB1 in the radioresponse of NPC. Colony formation and cell survival results showed that BPIFB1 sensitized NPC cells to ionizing radiation. VTN, a previously identified BPIFB1-binding protein, was shown to induce cell proliferation and survival, G2/M phase arrest, DNA repair, activation of the ATM-Chk2 and ATR-Chk1 pathways, and anti-apoptotic effects after exposure to radiation, facilitating NPC cell radioresistance. However, BPIFB1 inhibited this VTN-mediated radioresistance, ultimately improving NPC radiosensitivity. In conclusion, this study is the first to demonstrate the functions of BPIFB1 and VTN in the NPC radioresponse. Our findings indicated that promoting BPIFB1 expression and targeting VTN might represent new therapeutic strategies for NPC.

## Introduction

Nasopharyngeal carcinoma (NPC) is a distinct type of head and neck malignancy associated with remarkable geographic and racial differences^1,2^; it is rare in most parts of the world, but occurs relatively frequently in Southeast Asia and southern China^3^. Moreover, the occurrence of NPC continues to increase in individuals who migrate to Western countries from these areas^4^. NPC is relatively sensitive to ionizing radiation (IR), and thus radiotherapy is routine and the only curative treatment for this type of cancer^5^. Although radiotherapy can control local NPC and is associated with a positive outcome in early stages, a high proportion of patients still experience radiation resistance, which is the major cause of local recurrence and distant metastasis, resulting in treatment failure^6,7^. However, the malignant behavior of residual cells after irradiation and the associated underlying mechanisms are still unclear. Therefore, further studies on the molecular mechanisms of NPC radioresistance will improve anti-cancer therapy and prognosis for patients with NPC.

Bactericidal/permeability-increasing (BPI)-fold-containing family B member 1 (BPIFB1), also known as long-palate lung and nasal epithelium clone 1 (LPLUNC1), belongs to the BPI-fold-containing family^8^. Our previous study found that it is specifically expressed in nasopharyngeal epithelia and downregulated in NPC tissues^9^. BPIFB1 delays NPC cell growth, reduces NPC metastasis and invasion, and significantly inhibits interleukin-6 (IL-6)-induced NPC cell proliferation by decreasing signal transducer and activator of transcription 3 (STAT3) activation^10–12^. In addition, BPIFB1 expression in NPC tissues is positively associated with patient survival, indicating that reduced BPIFB1 expression constitutes a novel adverse prognostic factor for NPC^11^. Moreover, BPIFB1-positive NPC is associated with longer progression-free survival and overall survival compared to that of BPIFB1-negative NPC in similarly classified patients administered the same dose of radiotherapy^11^. This suggests that BPIFB1 might be associated with radiotherapy sensitivity in NPC. However, this role has not been clarified.

In the present study, we verified the hypothesis that re-expression of BPIFB1 can enhance the radiosensitivity of CNE2 and HONE1 cells in vitro. Furthermore, we found that BPIFB1 regulated the NPC cell radioresponse by inhibiting the expression of the BPIFB1-binding protein VTN. Specifically, ectopic expression of BPIFB1 inhibited VTN-induced anti-apoptotic effects; cell cycle arrest; DNA double-strand break (DSB) repair; and the activation of DSB repair-associated pathways, including ataxia telangiectasia mutated kinase-Chk2 (ATM-Chk2) and ataxia telangiectasia and Rad3-related kinase-Chk1 (ATR-Chk1); in addition to suppressing NPC cell radioresistance, which ultimately improved radiosensitivity.

## Results

### Re-expression of BPIFB1 sensitized NPC cells to IR

**Fig. 1:**
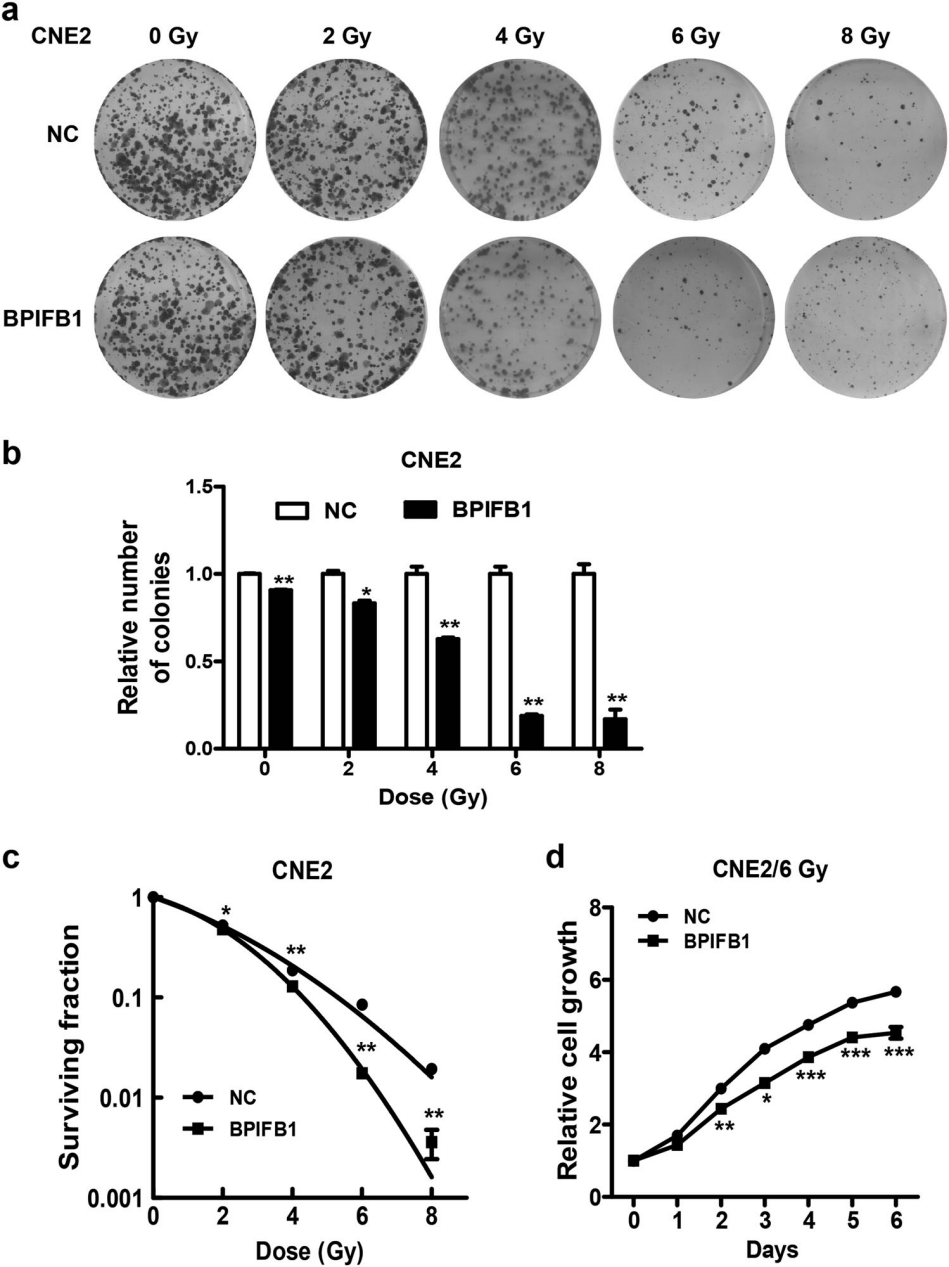
BPIFB1 re-expression sensitized CNE2 nasopharyngeal carcinoma (NPC) cells to ionizing radiation (IR). **a** Effects of BPIFB1 on clone formation ability of CNE2 cells after irradiation with a dose of 0–8 Gy. **b** Numbers of surviving foci are presented as bar graphs representing means ± SD. **c** Effects of BPIFB1 on survival rate of CNE2 cells after radiotherapy. Surviving fractions were calculated as described based on the data from experiments depicted in **b**. **d** CCK8 assays were used to investigate the role of BPIFB1 in CNE2 cell proliferation before and after irradiation. *P < 0.05; **P < 0.01; ***P < 0.001

Our previous study showed that lower levels of BPIFB1 were correlated with poor prognosis in patients with NPC^11^. To further identify the effect of BPIFB1 on the radioresponse of NPC cells, CNE2 and HONE1 cells were transfected with empty or BPIFB1 overexpression vectors, and then treated in a single session with doses of 0, 2, 4, 6, and 8 Gy. Colony formation assays were performed to determine radiosensitivity. The results showed that the ability to form survival foci in NPC cells was significantly suppressed by BPIFB1 overexpression, and this inhibition was particularly pronounced at 4 and 6 Gy in CNE2 (Fig. 1a, b) and HONE1 (Supplementary Fig. 1a, b) cells. Cell survival curves indicated that BPIFB1 overexpression resulted in better survival compared to that of control cells (Fig. 1c and Supplementary Fig. 1c). In addition, the effect of BPIFB1 on cell proliferation in response to irradiation was determined by performing Cell Counting Kit-8 (CCK8) assays. As shown in Fig. 1d and Supplementary Fig. 1d, BPIFB1 overexpression inhibited NPC cell proliferation after 6 Gy of irradiation as a single dose. Taken together, the aforementioned data showed that overexpression of BPIFB1 enhanced NPC cell radiosensitivity.

### BPIFB1 regulated the NPC cell radioresponse via VTN

In our previous study, co-immunoprecipitation combined with mass spectrometry analysis was performed to identify the BPIFB1-interacting protein VTN, which is a component of the extracellular matrix. BPIFB1 can bind VTN and suppress its expression and the formation of the VTN/integrin αV (ITGAV, a receptor of VTN) complex^12^. VTN is a multi-functional adhesive glycoprotein; however, its potential function in the radioresponse is still relatively unknown. Thus, we investigated the role of this protein in the NPC radioresponse to further explore whether BPIFB1 can enhance NPC cell radiosensitivity by regulating VTN expression.

**Fig. 2:**
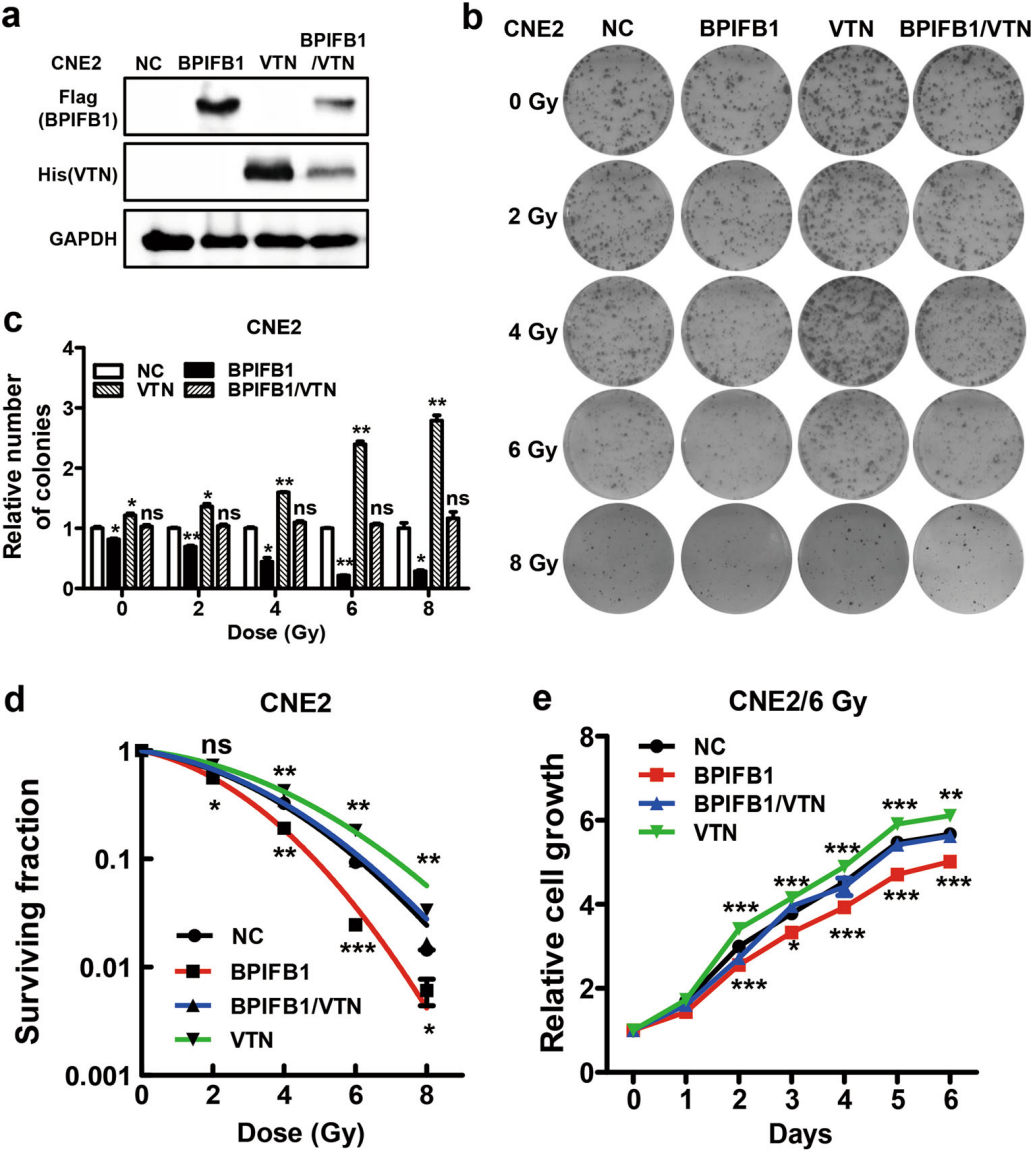
BPIFB1 regulated the CNE2 nasopharyngeal carcinoma cell radioresponse by interacting with VTN. **a** BPIFB1 and VTN expression were confirmed by western blotting in CNE2 cells transfected or co-transfected with BPIFB1-Flag and VTN-His vectors, using anti-Flag and anti-His primary antibodies. **b** Clone formation assay showing the response of CNE2-NC (negative control), CNE2-BPIFB1, CNE2-VTN, and CNE2-BPIFB1/VTN cells to 0–8 Gy radiotherapy. **c** The numbers of surviving foci in the four groups are presented as bar graphs representing means ± SD. **d** Effects of BPIFB1 and VTN on survival of CNE2 cells after radiotherapy. Surviving fractions were calculated as described based on the data from experiments in **c**. **e** CCK8 assays were used to investigate the role of BPIFB1 and VTN in CNE2 cell proliferation after irradiation. *P *<* 0.05; **P *<* 0.01; ***P *<* 0.001; NS, no significance

Colony formation assays showed that BPIFB1 inhibited the survival and foci formation of cells exposed to IR, whereas VTN expression enhanced the colony-forming ability of CNE2 (Fig. 2a, b) and HONE1 (Supplementary Fig. 2a, b) cells. Notably, BPIFB1 reversed the effect of VTN when the proteins were co-expressed in CNE2 cells (Fig. 2b, c) and HONE1 cells (Supplementary Fig. 2b, c). In contrast to BPIFB1, VTN significantly enhanced the fraction of surviving cells, indicating higher radioresistance. However, BPIFB1 attenuated VTN-induced radioresistance in the two cell lines (Fig. 2d and Supplementary Fig. 2d). Furthermore, the role of BPIFB1 and VTN in NPC cell radioresponse was investigated after 6 Gy as a single dose or as multiple doses (a daily dose of 2 Gy for 3 days). We observed the same results in both radiation delivery schemes, indicating that BPIFB1 inhibited VTN-induced radioresistance of NPC cells. The inhibitory effect was more pronounced from a single dose of IR than from fractionated doses (Supplementary Fig. 3a, b). Thus, the NPC cells were treated in a single session with a dose of 6 Gy in the follow-up experiments.

The effect of VTN on cell growth was then examined by treating CNE2 and HONE1 cells with a single radiation dose of 6 Gy. As shown in Fig. 2e and Supplementary Fig. 2e, transfection with VTN increased cell numbers as compared to transfection with control vectors after treatment with 6 Gy of IR. This increase was dramatically attenuated by BPIFB1 co-expression. Collectively, these data demonstrated that VTN-regulated NPC cell radioresistance is mediated by BPIFB1 expression, and that BPIFB1 overexpression promotes NPC cell radiosensitivity by downregulating VTN.

### Overexpression of BPIFB1 inhibited VTN-induced anti-apoptotic effects after irradiation

**Fig. 3:**
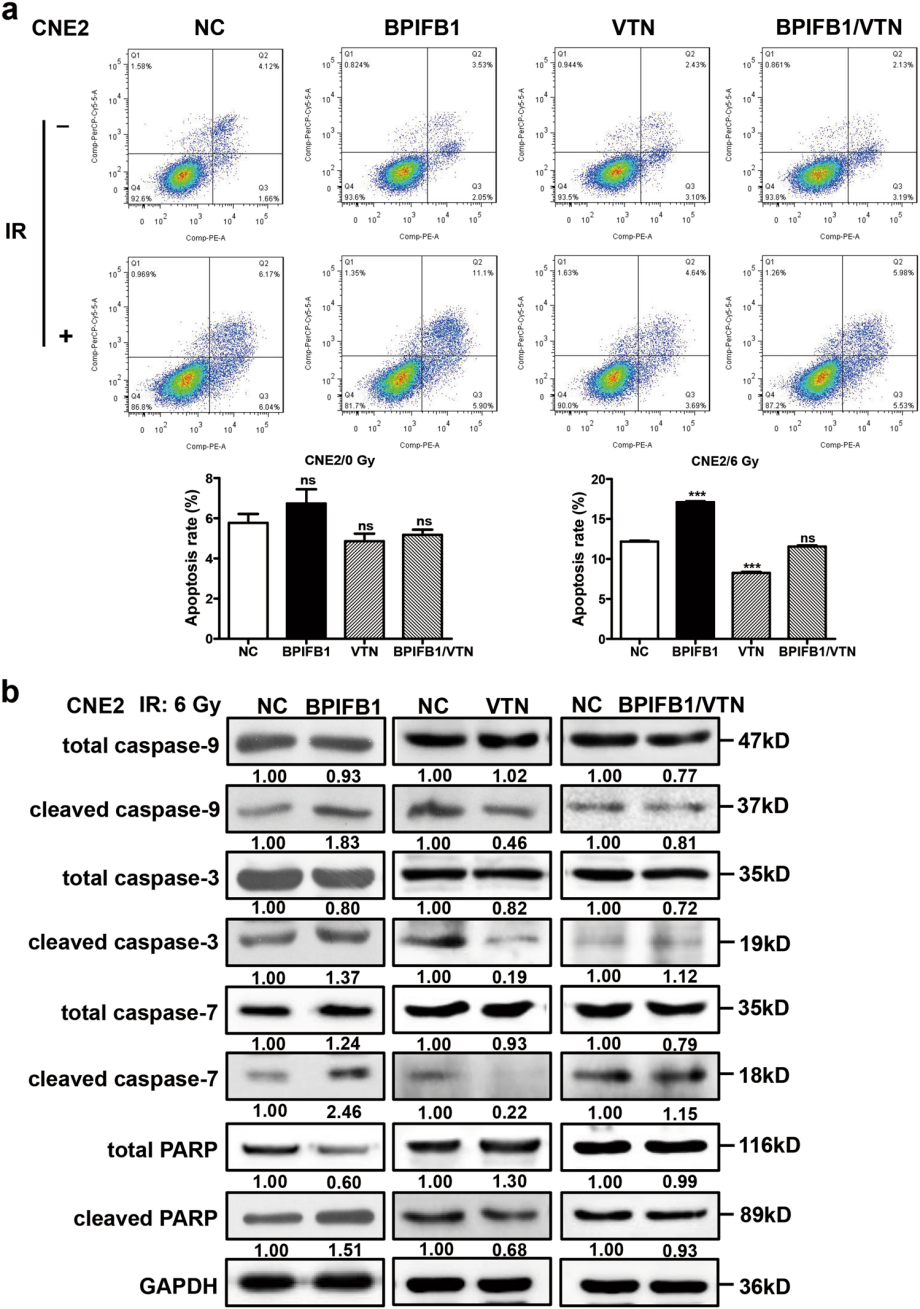
Overexpression of BPIFB1 inhibited VTN-induced anti-apoptotic effects in CNE2 nasopharyngeal carcinoma cells after irradiation. **a** (Top) BPIFB1-overexpressing cells are sensitive to IR-induced cell death. The four groups of CNE2 cells were treated with or without 6 Gy of ionizing radiation and cells were stained with annexin V to measure the percentage of apoptotic cells. (Below) The percentage of apoptotic cells is presented as bar graphs representing means ± SD. ***P *<* 0.001; NS, no significance. **b** Expression of typical apoptosis markers, including cleaved caspase-9, cleaved caspase-3, cleaved caspase-7, and cleaved PARP, based on western blotting, comparing the four groups of CNE2 cells transfected or co-transfected with the BPIFB1-Flag and VTN-His vectors. GAPDH was used as an internal control. NC negative control. The numbers below blots represent grayscale values of each blot. The molecular weights of blots are indicated to their right

To investigate whether the BPIFB1-mediated decrease in NPC cell radioresistance was due to apoptosis, flow cytometry was performed. NPC cells were transfected or co-transfected with BPIFB1 and VTN overexpression vectors and subjected to IR. The results showed that BPIFB1 and VTN had no significant effect on NPC cell apoptosis before IR. However, 24 h after treating the cells with 6 Gy of IR, apoptotic rates increased in each group. In particular, the rate of apoptosis in BPIFB1-overexpressing cells was significantly increased compared to that in control cells. In contrast, VTN-overexpressing cells were more resistant to IR-induced apoptosis, and this was attenuated by BPIFB1 co-expression in CNE2 (Fig. 3a) and HONE1 (Supplementary Fig. 4a) cells.

Caspases are central regulators of apoptosis; caspase-9 is an important initiator, whereas caspase-3 and caspase-7 are crucial executioners^13^. Thus, we next detected protein levels of cleaved caspase-3, -7, and -9, as well as their activated substrate, cleaved poly-ADP ribose polymerase (PARP), 48 h after administering 6 Gy of IR. Consistently with the results of the apoptosis assay, the levels of all these markers were strongly increased in CNE2 and HONE1 cells transfected with the BPIFB1 overexpression vector. Overexpression of VTN decreased the activation of these proteins, and this was reversed by BPIFB1 co-expression (Fig. 3b and Supplementary Fig. 4b). These data suggested that overexpression of BPIFB1 led to the activation of apoptotic pathway proteins after irradiation, consequently inhibiting VTN-induced resistance to apoptosis and thereby enhancing the radiosensitivity of NPC cells.

### BPIFB1 impaired VTN-induced cell cycle arrest after irradiation

**Fig. 4:**
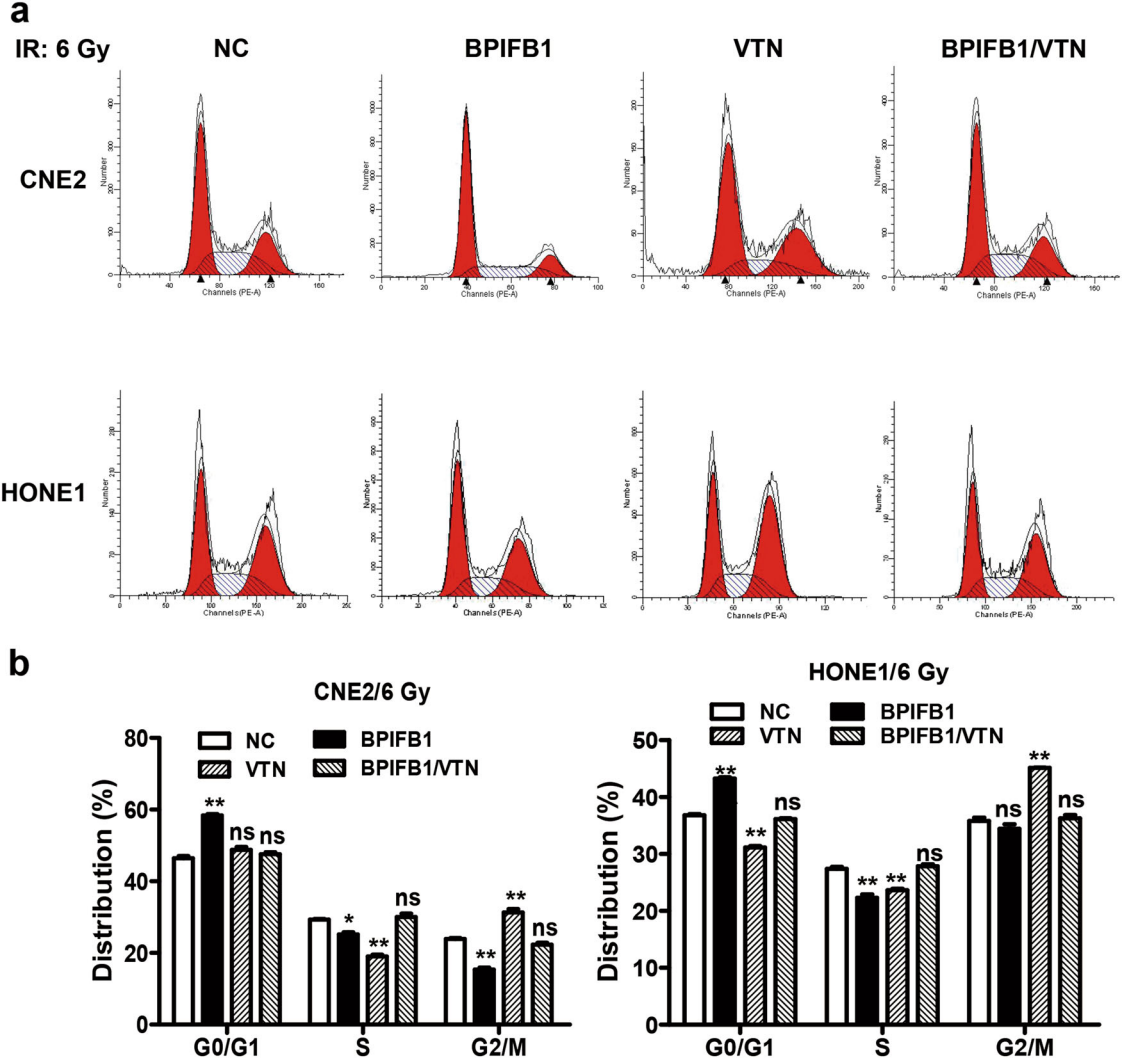
Overexpression of BPIFB1 impaired VTN-induced nasopharyngeal carcinoma cell cycle arrest after irradiation. Flow cytometry was performed to assess variations in cell cycle distribution. **a** BPIFB1 overexpression resulted in G0/G1 phase arrest, and VTN overexpression resulted in G2/M phase arrest in CNE2 and HONE1 cells after irradiation. The four groups of cells (overexpressing BPIFB1, VTN, both, and the control group) were incubated for 24 h after treatment with 6 Gy of radiation. **b** Histogram of cell cycle distribution of CNE2 and HONE1 cells. Data represent means ± SD from of at least three independent experiments; *P < 0.05; **P < 0.01; NS, no significance

To further investigate the underlying mechanism associated with BPIFB1-induced radiosensitivity in NPC cells, flow cytometry was performed to assess variations in cell cycle distribution. It has been reported that NPC cells exhibiting G1 arrest are more sensitive to IR than are those lacking this property^14,15^. Consistently, BPIFB1 led to a substantial increase in the proportion of CNE2 cells in G0/G1 phase and a concomitant decrease in the proportion of cells in the S and G2 phases (Fig. 4a). Moreover, the proportion of cells arrested at the G2/M phase in VTN-overexpressing CNE2 cells was dramatically higher than that in control cells, indicating that VTN participates in the G2/M checkpoint, which is beneficial for DNA damage repair and critical to preventing cell death^16^. However, VTN-induced G2/M phase arrest was restored to a level comparable to that of the control when BPIFB1 was co-expressed (Fig. 4b). Similarly, flow cytometric analyses using HONE1 cells showed the same cell cycle distribution (Fig. 4a, b). These results indicated that BPIFB1 promotes radiosensitivity by enhancing the G0/G1 phase arrest and inhibiting VTN-induced G2/M phase arrest after IR.

### BPIFB1 repressed VTN-induced DSB repair in NPC cells after irradiation

**Fig. 5:**
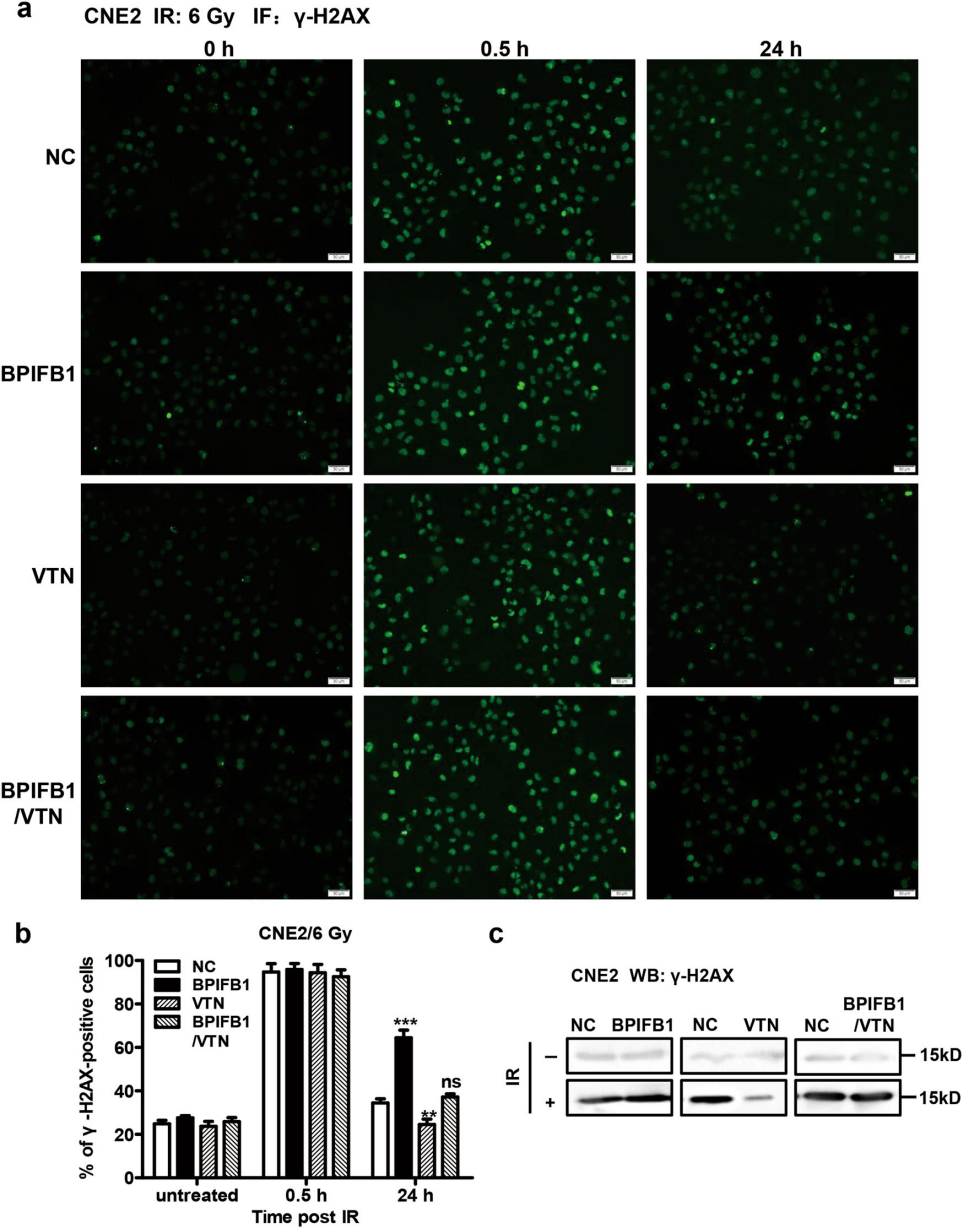
BPIFB1 repressed VTN-induced DNA repair in CNE2 nasopharyngeal carcinoma cells after irradiation. The levels of γ-H2AX at different times after 6 Gy irradiation were detected by immunofluorescence in CNE2 cells transfected or co-transfected with BPIFB1-Flag and VTN-His vectors. Cells displaying 10 or more foci were counted as positive. **a** Representative images of γ-H2AX foci in CNE2-NC (negative control), CNE2-BPIFB1, CNE2-VTN, and CNE2-BPIFB1/VTN cells are shown. **P *<* 0.01; ***P *<* 0.001; NS, no significance. Scale bar = 50 μm. **b** Histogram of the percentage of γ-H2AX foci in the four groups of CNE2 cells. **c** Detection of γ-H2AX protein levels in the four groups of CNE2 cells treated with or without 6 Gy of IR. The molecular weights of blots are indicated to their right

Within minutes of IR-induced DSBs, Ser139 of H2AX (a histone H2A family member) is rapidly phosphorylated around the DSB site, after which it is referred to as anti-phospho-histone H2AX (γ-H2AX), a recognized marker of DSBs positively associated with radiosensitivity^17^. Thus, immunofluorescent staining was performed to quantify DSBs by assessing the formation of γ-H2AX foci, using CNE2 cells overexpressing BFIFB1, VTN, or both, and comparing the results to those of control cells. The proportions of γ-H2AX-positive cells were very low in all four groups before exposure to IR. At 0.5 h after irradiation, robust formation of γ-H2AX foci was observed in all four groups at virtually equal levels. At 24 h post irradiation, the percentage of γ-H2AX-positive cells was much higher in cells overexpressing BPIFB1, compared to that in control cells, whereas the percentage of γ-H2AX foci in VTN-overexpressing cells was lower than that in control cells, indicating that most DSBs were repaired upon VTN overexpression. However, γ-H2AX foci formation in cells overexpressing both markers was comparable to that in control cells (Fig. 5a, b).

The protein level of γ-H2AX was also quantified by western blotting. Before IR, γ-H2AX levels were very low, and there were no differences among the four groups; levels increased in all groups after exposure to 6 Gy of IR. Compared to control cells, BPIFB1-overexpressing cells had higher levels, and VTN-overexpressing cells had the lowest levels of γ-H2AX (Fig. 5c), which was consistent with the results of the immunofluorescence assay. Moreover, consistent results were observed in HONE1 cells (Supplementary Fig. 5a–c), supporting the notion that BPIFB1 impairs VTN-induced DSB repair in NPC cells after irradiation.

### BPIFB1 overexpression inhibited VTN-induced activation of the ATM-Chk2 and ATR-Chk1 pathways after IR

**Fig. 6:**
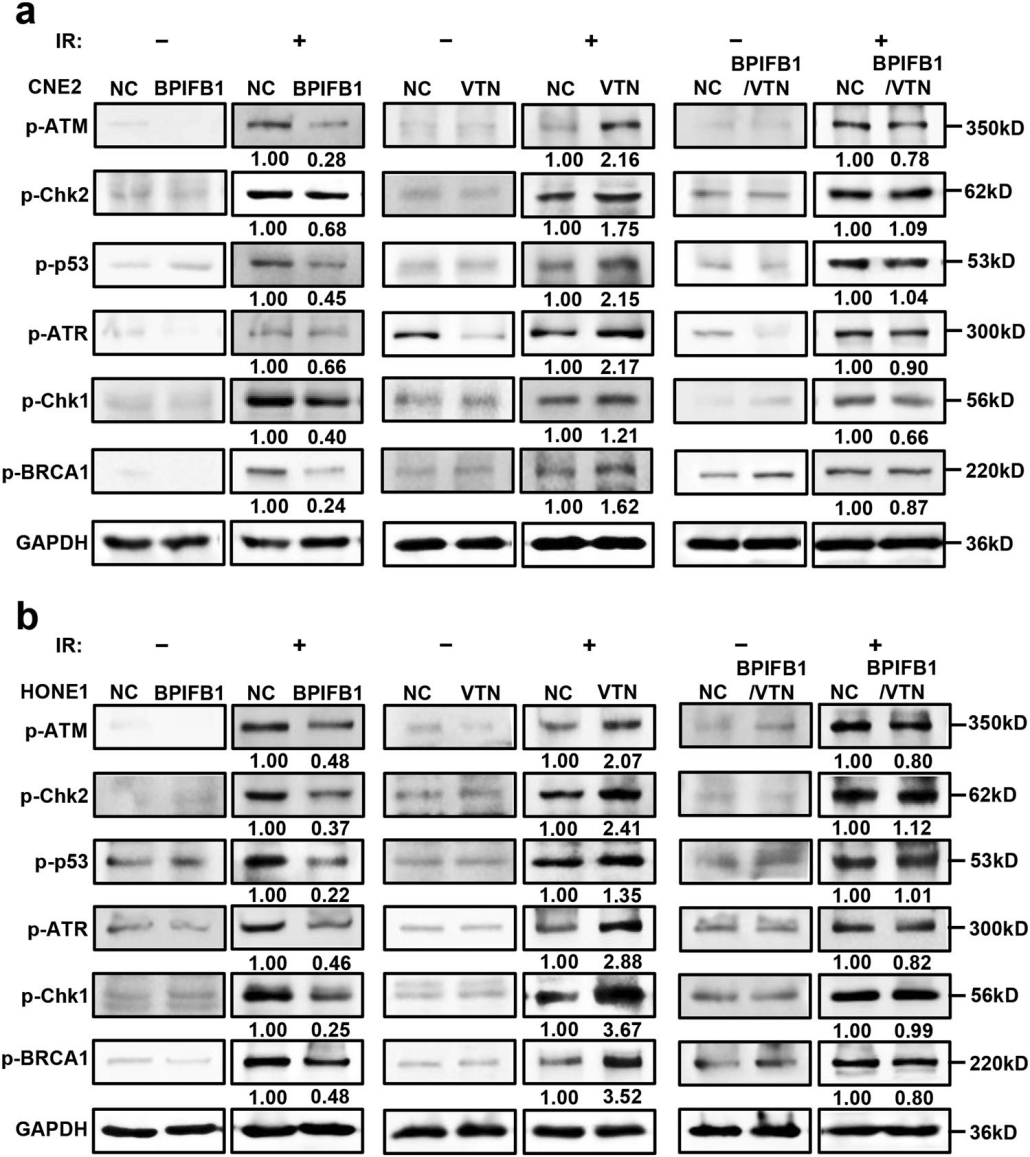
Effects of BPIFB1 and VTN on ionizing radiation (IR)-induced activation of the ATM/Chk2 and ATR/Chk1 pathways in nasopharyngeal carcinoma (NPC) cells. Expression of ATM/Chk2 and ATR/Chk1 pathway-associated proteins. Protein levels of phospho (p)-ATM, p-Chk2, p-p53, p-ATR, p-Chk1, and p-BRCA1 were detected by western blotting in **a** CNE2 cells and **b** HONE1 cells transfected or co-transfected with BPIFB1-Flag and VTN-His vectors. NC, negative control. The numbers below blots represent grayscale values of each blot. The molecular weights of blots are indicated to their right

Upon IR-induced DNA damage, tumor cells utilize two primarily distinct kinase signaling cascades to repair DSBs, including the ATM-Chk2 and ATR-Chk1 axes^18^. To investigate the role of BPIFB1 and VTN in the activation of these pathways after irradiation, we assessed the phosphorylation of ATM (p-ATM), Chk2 (p-Chk2), p53 (p-p53), ATR (p-ATR), Chk1 (p-Chk1), and BRCA1 (p-BRCA1) in CNE2 and HONE1 cells before and after irradiation. As shown in Fig. 6a, b, in non-irradiated cells, the phosphorylation levels of these proteins were extremely low and not significantly different among the four groups of cells. After administering a radiation dose of 6 Gy, the activation of these proteins increased. Moreover, compared to those in control cells, the phosphorylation levels of ATM, Chk2, p53, ATR, Chk1, and BRCA1 were significantly decreased with BPIFB1 overexpression, whereas VTN-overexpressing cells exhibited relatively higher activation of these two pathways. However, when BPIFB1 and VTN were co-expressed in NPC cells, the increased phosphorylation of these proteins was almost restored to control levels. Thus, our data showed that overexpression of BPIFB1 repressed the VTN-induced activation of ATM-Chk2 and ATR-Chk1 pathways after IR, and consequently decreased the DNA repair ability of NPC cells, ultimately enhancing radiosensitivity.

## Discussion

Our previous study suggested that BPI-fold-containing family, previously known as the PLUNC protein family, is mainly expressed in the human nasopharynx, trachea, lung, and salivary glands^19,20^. Notably, members of this family all contain domains structurally similar to lipopolysaccharide-binding protein and BPI. Thus, this family comprises the “epithelial frontier,” owing to its host defense and innate immune properties^21^. In our previous study, suppression subtractive hybridization and cDNA microarray assays were performed to identify a nasopharyngeal epithelial tissue-specific gene, BPIFB1, which is significantly downregulated in NPC^9^. BPIFB1 is a potential tumor suppressor that regulates NPC cell growth by downregulating the MAPK and cyclin D1/E2F pathways^10^. Moreover, BPIFB1 suppresses IL-6-induced NPC cell proliferation by inhibiting STAT3 activation^11^. In addition, this protein can inhibits NPC cell metastasis and invasion^12^. However, there has been no report on the relationship between BPIFB1 and radiation sensitivity.

NPC is a cancer arising from the nasopharynx epithelium and has a very unique pattern of geographical distribution^4,22^. Unlike other head and neck cancers, this disease is strongly associated with Epstein–Barr virus infection^3,23^. The location of NPC is deep and secluded, and early symptoms are atypical. More than 90% of NPC is poorly differentiated squamous cell carcinoma that is moderately radiosensitive, and thus radical radiation therapy is the first choice of treatment^6^. BPIFB1 expression is positively correlated with NPC patient survival rate^11^; thus, we hypothesized that BPIFB1 might sensitize NPC cells to IR. To test this hypothesis, in vitro radioresponse assays were performed after overexpressing BPIFB1 in NPC cells. Considering that endogenous expression of BPIFB1 was very low in these cells, the loss-of-function approach was not performed. We found that BPIFB1 significantly inhibited the formation of survival foci and proliferation in CNE2 and HONE1 cells, indicating that BPIFB1 is capable of enhancing NPC cell radiosensitivity.

Next, we investigated the underlying mechanism of the BPIFB1-regulated NPC radioresponse. We previously identified a BPIFB1-interacting protein, VTN^12^. As an extracellular matrix protein, VTN is a multi-functional adhesive glycoprotein that participates in many physiological and pathological processes^24^. VTN can affect tumor cell adhesion, motility, invasion, and proliferation, in addition to protecting tumor cells from apoptosis-related cell death^25,26^. However, the role of VTN in radioresistance remains unclear. We therefore speculated that VTN might play a critical role in NPC cell radioresistance. As expected, overexpression of VTN significantly increased radioresistance in NPC cells, and this resistance was dramatically inhibited upon BPIFB1 co-expression. This is the first evidence suggesting that VTN modulates NPC radioresistance. Together, these results demonstrate that BPIFB1 is capable of inhibiting VTN-induced radioresistance in NPC cells.

As the receptor of ITGAV, VTN can induce integrin clustering and positively regulate ITGAV expression^27^. Our previous research showed that BPIFB1 interacts with this protein, inhibits expression of VTN and ITGAV, and consequently reduces VTN-ITGAV complex formation and the activation of the downstream FAK/Src/ERK pathway, ultimately enhancing NPC cell invasion and migration^12^. It has been reported that ITGAV induces radioresistance by activating the SAPK/JNK pathway^28^. Owing to the inhibitory effect on the formation of VTN-ITGAV complexes and the activation of the FAK pathway, we speculated that BPIFB1 inhibits the VTN-ITGAV-FAK pathway, ultimately enhancing NPC cell radiosensitivity.

Apoptosis is an important mechanism through which IR exerts its therapeutic potential, and apoptosis resistance in cancer cells is considered the main cause of radiotherapy failure and radioresistance^29^. Thus, we evaluated whether BPIFB1-induced radiosensitivity and VTN-induced radioresistance might affect NPC cell apoptosis after IR. Our results suggested that BPIFB1-VTN signaling indeed affects this process. Specifically, VTN overexpression increased resistance to IR-induced apoptosis, but this effect was attenuated upon BPIFB1 co-expression in NPC cells. Caspases, a family of cysteine acid proteases, are important to the regulation of apoptosis^30^. They are initially produced as inactive monomeric procaspases that require dimerization and often cleavage for activation^13^. Caspase-9, an important initiator caspase, has the ability to couple proapoptotic signals. Once activated, caspase-9 cleaves and activates downstream executioner caspases, including caspase-3 and caspase-7^31^. Subsequently, PARP, a well-known DNA-binding enzyme, is cleaved and activated, triggering apoptosis^32^. In this study, we found that VTN significantly down-regulated cleaved caspase-9, cleaved caspase-3, cleaved caspase-7, and cleaved PARP in NPC cells after radiotherapy, whereas BPIFB1 reversed this inhibitory effect. This indicated that BPIFB1 promotes the expression of apoptosis-associated proteins, induces cell death, and improves NPC cell radiosensitivity.

When IR-induced DSBs occur, checkpoints regulate and arrest the cell cycle in response to DNA damage; these play a crucial role in determining radioresistance^15^. To date, many publications have indicated that increased G0/G1 arrest is associated with radiosensitivity, whereas G2/M arrest is associated with radioresistance^14,16,33^. In this study, our results showed that BPIFB1 induced a significant increase in G0/G1 arrest after irradiation, which strongly supported our observation that BPIFB1 enhanced radiosensitivity by inducing G0/G1 arrest in NPC cells. In contrast, VTN was found to induce the accumulation of cells in the G2/M phase after radiation treatment; however, BPIFB1 co-expression reversed this effect. The arrest of cells at the G2/M phase means that cell mitosis has been delayed, providing sufficient time for DSB repair^34–36^. Thus, BPIFB1 inhibits VTN-induced G2/M arrest, and consequently decreases DNA repair, increases cell death, and finally reduces NPC cell radioresistance.

Radiometric-induced DSBs are the most lethal form of DNA damage, and the ability to repair DSBs is closely associated with sensitivity to radiotherapy^17^. Thus, enhanced DSB repair is a critical mechanism through which cells can become resistant to IR^37^. As stated, during the early stage of DSB repair, Ser139 of H2AX is rapidly phosphorylated, and within minutes following DNA damage, this form (γ-H2AX) localizes to sites of DNA damage at subnuclear foci^38^. Therefore, the level of γ-H2AX is positively associated with radiosensitivity. Our study demonstrated that BPIFB1 induced increased levels of γ-H2AX, indicating the presence of unrepaired DSBs. Moreover, VTN-overexpressing NPC cells exhibited decreased γ-H2AX levels, indicating enhanced DSB repair. These data indicated that BPIFB1 represses VTN-induced DSB repair in NPC cells.

When sensing IR-induced DNA damage in NPC cells, there are two main pathways that rapidly respond and initiate DNA repair, namely, ATM-Chk2 and ATR-Chk1^18^. ATM and ATR are PI3 kinase-related proteins that phosphorylate multiple substrates on serine or threonine residues in response to DNA damage^39^. Under such conditions, Ser1981 on ATM is rapidly auto-phosphorylated; it can then activate ATM, which phosphorylates the downstream effector kinase-Chk2 on Thr68. This is followed by the phosphorylation of p53 on Ser15^40^. Phosphorylation of p53 inhibits its interaction with MDM2, resulting in enhanced p53 transcriptional activity, which promotes the activation of target genes such as p21, ultimately causing G2 phase arrest and effective DNA repair without apoptosis^41,42^. Moreover, Chk1, a downstream protein kinase of ATR, can be phosphorylated on Ser345. Additionally, ATM-Chk2 and ATR-Chk1 are involved in the activation of BRCA1, which has a role in processes related to DNA repair^18,43^. In the present study, we found that overexpression of VTN enhanced DNA damage repair by activating both ATM-Chk2 and ATR-Chk1 pathways, and that BPIFB1 inhibited this effect, thereby reducing G2/M phase arrest and leading to radiosensitivity.

In summary, our study demonstrated that BPIFB1 is involved in the modulation of radiosensitivity in NPC. In particular, BPIFB1 negatively regulates its interactor VTN, thereby inhibiting VTN-induced proliferation, anti-apoptotic effects, G2/M phase arrest, DSB repair, and the activation of the ATM-Chk2 and ATR-Chk1 pathways after irradiation; this ultimately improved NPC cell radiosensitivity. To our knowledge, this represents the first study demonstrating the functions of BPIFB1 and VTN in the NPC radioresponse, indicating that BPIFB1 might represent a good biomarker for prognosis and a novel therapeutic target for increasing the radiosensitivity of NPC. In addition, targeting VTN might represent a new therapeutic strategy for NPC treatment.

## Materials and methods

### Cell lines, expression constructs, and transfection

The NPC cell lines CNE2 and HONE1 were maintained in our laboratory and grown in RPMI-1640 medium (Life Technologies, Carlsbad, CA, USA) supplemented with 10% fetal bovine serum (Life Technologies) and 1% penicillin–streptomycin (Life Technologies). Cells were incubated at 37 °C in a humidified atmosphere with 5% CO_2_.

To generate the BPIFB1 overexpression vectors, the full-length BPIFB1-coding sequence was amplified, tagged with Flag, cloned into the pIRESneo3 plasmid (Life Technologies), and named pIRESneo3-BPIFB1/Flag (BPIFB1-Flag). The full-length VTN coding sequence was also amplified and cloned into the pcDNA6-His vector (Life Technologies), generating pcDNA6-His/VTN (VTN-His). Transfection was performed using Lipofectamine 3000 reagent (Life Technologies) according to the manufacturer’s protocol.

### Cell radiation assays

For colony formation assays, cells were seeded in 6-well plates after transfection with BPIFB1-Flag or VTN-His vectors; they were then incubated for 24 h for seeding. Cells were treated in a single session with radiation doses of 2, 4, 6, and 8 Gy. Approximately 7–10 days later, colonies were stained with 0.5% crystal violet and counted using the standard definition that a colony consists of 50 or more cells. The dose survival curve was plotted using the linear quadratic model (*Y* = exp(−(a ×* x* + *b *× (*x*^2^)))).

For cell proliferation assays, 800 CNE2 cells were seeded in 96-well plates after transfection. CCK8 reagent (DOHINDO, Kumamoto, Japan) was added to each well to measure the number of viable cells in each well at 0, 1, 2, 3, 4, 5, and 6 days. Optical density was measured at a wavelength of 450 nm (OD_450_).

### Western blotting

Whole-cell lysates were harvested using radioimmunoprecipitation assay buffer with a protease inhibitor cocktail and the PhosSTOP phosphatase inhibitor (Roche, Basel, Switzerland), and protein concentrations were determined using the BCA assay (Pierce, Dallas, TX, USA). Protein samples were then subjected to 8–12% sodium dodecyl sulfate-polyacrylamide gel electrophoresis separation and blotted onto polyvinylidene fluoride membranes (EMD Millipore, Billerica, MA, USA). The membrane was blocked with 5% dry skim milk and then incubated with the following primary antibodies at 4 °C overnight: anti-Flag (Sigma-Aldrich, St. Louis, MO, USA), His, phospho-ATR (Ser428), phospho-ATM (Ser1981), phospho-Chk1 (Ser345), phospho-Chk2 (Thr68), phospho-BRCA1 (Ser1524), phospho-p53 (Ser15), PARP, cleaved PARP, caspase-3, cleaved caspase-3, caspase-7, cleaved caspase-7, caspase-9, cleaved caspase-9 (Cell Signaling Technology, Danvers, MA, USA). After washing, the blots were incubated with horseradish peroxidase-labeled secondary antibodies (Cell Signaling Technology) and detected by enhanced chemiluminescence (EMD Millipore). GAPDH (Cell Signaling Technology) served as an endogenous loading control. Full gel images for all western blot experiments are provided in Supplementary Fig. 6.

### Immunofluorescence assay

Cells were seeded on coverslips in a 6-well plate, and then exposed to a radiation dose of 6 Gy. After culturing for specified times (0, 0.5, and 24 h), cells were fixed in 4% paraformaldehyde for 20 min, permeabilized with 0.25% Triton X-100 for 40 min, and blocked in 5% bovine serum albumin for 1 h. Cells were immunostained with γ-H2AX (Cell Signaling Technology) at 4 °C overnight, followed by incubation with secondary fluorochrome-labeled antibody (Alexa Fluor 488 donkey anti-rabbit IgG (H + L) antibody; Life Technologies) for 40 min at 37 °C. The cells were then stained with 4′,6-diamidino-2-phenylindole to visualize nuclear DNA. Images were captured using an Olympus fluorescence microscope (Olympus Optical Co., Tokyo, Honshu, Japan).

### Flow cytometry

Cells transfected or co-transfected with BPIFB1-Flag and VTN-His were seeded in 6-well plates and irradiated with 6 Gy of X-rays; they were then incubated for 24 h after IR. For cell cycle assays, cells were fixed with 75% ethanol at 4 °C overnight and stained with propidium iodide (50 µg/ml) and RNase A (1 mg/ml). Cell cycle distribution was determined by a FACSCalibur Flow Cytometer (BD Biosciences, Franklin Lakes, NJ, USA) and analyzed using ModFit (Verity Software House, Topsham, ME, USA). For apoptosis assays, cells were trypsinized, washed, resuspended in 200 μl binding buffer, and then evaluated for apoptosis by double staining with 5 μl 7-aminoactinomycin D and 5 μl annexin V (all from BD Biosciences) using a FACSCalibur Flow Cytometer.

### Statistical analysis

All experiments were performed at least three times. Data are presented as means ± standard deviation (SD). Statistical analyses were performed using GraphPad Prism 5 (GraphPad Software, Inc., La Jolla, CA, USA). Student’s *t* tests or *χ*^2^ tests were performed to evaluate significant differences between two groups of data. *P < *0.05 was considered to indicate statistical significance.

## Electronic supplementary material

supplementary information

supplementary Figure 1

supplementary Figure 2

supplementary Figure 3

supplementary Figure 4

supplementary Figure 5

supplementary Figure 6-1

supplementary Figure 6-2
