# Supplementary material for: BPIFB1 (LPLUNC1) inhibits radioresistance in nasopharyngeal carcinoma by inhibiting VTN expression
Source: Cell Death Dis. 2018 Mar 22;9(4):432. doi: 10.1038/s41419-018-0409-0 (PMC5864881; doi:10.1038/s41419-018-0409-0)
Supplement: Supplementary file 1 — supplementary information [file 41419_2018_409_MOESM1_ESM.docx]

**Supplementary Information**

The Supplementary Information file contains Supplementary Fig. 1 (BPIFB1 sensitized HONE1 cells to ionizing radiation); Supplementary Fig. 2 (BPIFB1 regulated HONE1 cell radioresponse by interacting with VTN); Supplementary Fig. 3 (BPIFB1 inhibited VTN-induced radioresistance of CNE2 cells after 6 Gy as a single dose or a daily dose of 2 Gy for 3 d); Supplementary Fig. 4 (BPIFB1 inhibited VTN-induced anti-apoptotic effects in HONE1 cells after radiation); Supplementary Fig. 5 (BPIFB1 repressed VTN-induced DNA repair of HONE1 cells after ionizing radiation) ; Supplementary Fig. 6 (Full scans of all western blots).

**Supplementary Figure Legends**

**Supplementary Fig. 1 BPIFB1 re-expression sensitized HONE1 cells to ionizing radiation. a** Effects of BPIFB1 on clone formation ability of HONE1 cells after irradiation with doses of 0-8 Gy. **b** The numbers of surviving foci are presented as bar graphs representing means ± SD. **c** Effects of BPIFB1 on survival rate of HONE1 cells after radiotherapy; surviving fractions were calculated as described based on data from experiments in (b). **P* < 0.05; ***P*< 0.01; ****P* < 0.001.

**Supplementary Fig. 2 BPIFB1 regulated HONE1 cell radioresponse by interacting with VTN. a** BPIFB1 and VTN expression was confirmed by western blotting in HONE1 cells transfected or co-transfected with BPIFB1-Flag and VTN-His vectors using anti-Flag and anti-His primary antibodies. **b** Clone formation assays showed the response of HONE1-NC (negative control), HONE1-BPIFB1, HONE1-VTN, and HONE1-BPIFB1/VTN cells to 0-8 Gy radiotherapy. **c** The numbers of surviving foci in the four cell groups are presented as bar graphs representing means ± SD. **d** Effects of BPIFB1 and VTN on survival of HONE1 cells after radiotherapy; surviving fractions were calculated as described based on the data from experiments depicted in (c). **P* < 0.05; ***P* < 0.01; ****P* < 0.001; ns, no significance.

**Supplementary Fig. 3 BPIFB1 inhibited VTN-induced radioresistance of CNE2 cells after 6 Gy as a single dose or a daily dose of 2Gy for 3 d. a** Clone formation assay showing the response of CNE2-NC (negative control), CNE2-BPIFB1, CNE2-VTN, and CNE2-BPIFB1/VTN cells treated with IR as a single dose of 6 Gy or multiple doses (a daily dose of 2 Gy for 3 d). **b** The numbers of surviving foci in the four groups are presented as bar graphs representing means ± SD. **P* < 0.05; ***P* < 0.01; ****P* < 0.001; ns, no significance.

**Supplementary Fig. 4 Overexpression of BPIFB1 inhibited VTN-induced anti-apoptotic effects in HONE1 cells after ionizing radiation (IR). a** (Top) BPIFB1-overexpressing cells were sensitive to IR-induced cell death. Four groups of HONE1 cells (overexpression BPIFB1, VTN, both, or the negative control) were treated with or without 6 Gy of IR, and the cells were stained with annexin V to measure the percentage of apoptotic cells. (Below) The percentage of apoptotic cells is presented as bar graphs representing means ± SD. ***P* < 0.01; ****P* < 0.001; ns, no significance. **b** Expression of typical apoptosis markers, including cleaved caspase-9, cleaved caspase-3, cleaved caspase-7, and cleaved PARP, according to western blotting performed on the four groups of HONE1 cells transfected or co-transfected with the BPIFB1-Flag and VTN-His vectors. GAPDH was used as an internal control. NC: negative control. The numbers below blots represent grayscale values of each blot. The molecular weights of blots are indicated to their right.

**Supplementary Fig. 5 BPIFB1 repressed VTN-induced DNA repair in HONE1 cells after ionizing radiation (IR).** The levels of γ-H2AX at different times after 6 Gy of IR were detected by immunofluorescence using HONE1 cells transfected or co-transfected with BPIFB1-Flag and VTN-His vectors. Cells displaying 10 or more foci were counted as positive. **a** Representative images of γ-H2AX foci in HONE1-NC (negative control), HONE1-BPIFB1, HONE1-VTN, and HONE1-BPIFB1/VTN cells are shown. ****P* < 0.001; ns, no significance. Scale bar = 50 μm. **b** Histogram of the percentages of γ-H2AX foci in the four groups of HONE1 cells (overexpressing BPIFB1, VTN, both, or the negative control). **c** Detection of γ-H2AX protein levels in the four groups of HONE1 cells treated with or without 6 Gy of IR. The molecular weights of blots are indicated to their right.

**Supplementary Fig. 6 Full scans of all western blots.** The red boxes showed the corresponding bands in all figures, and the order of the bands is the same as the original figure (except for some specially labeled ones).
