# Supplementary figures and images for: BPIFB1 (LPLUNC1) inhibits radioresistance in nasopharyngeal carcinoma by inhibiting VTN expression
Source: Cell Death Dis. 2018 Mar 22;9(4):432. doi: 10.1038/s41419-018-0409-0 (PMC5864881; doi:10.1038/s41419-018-0409-0)

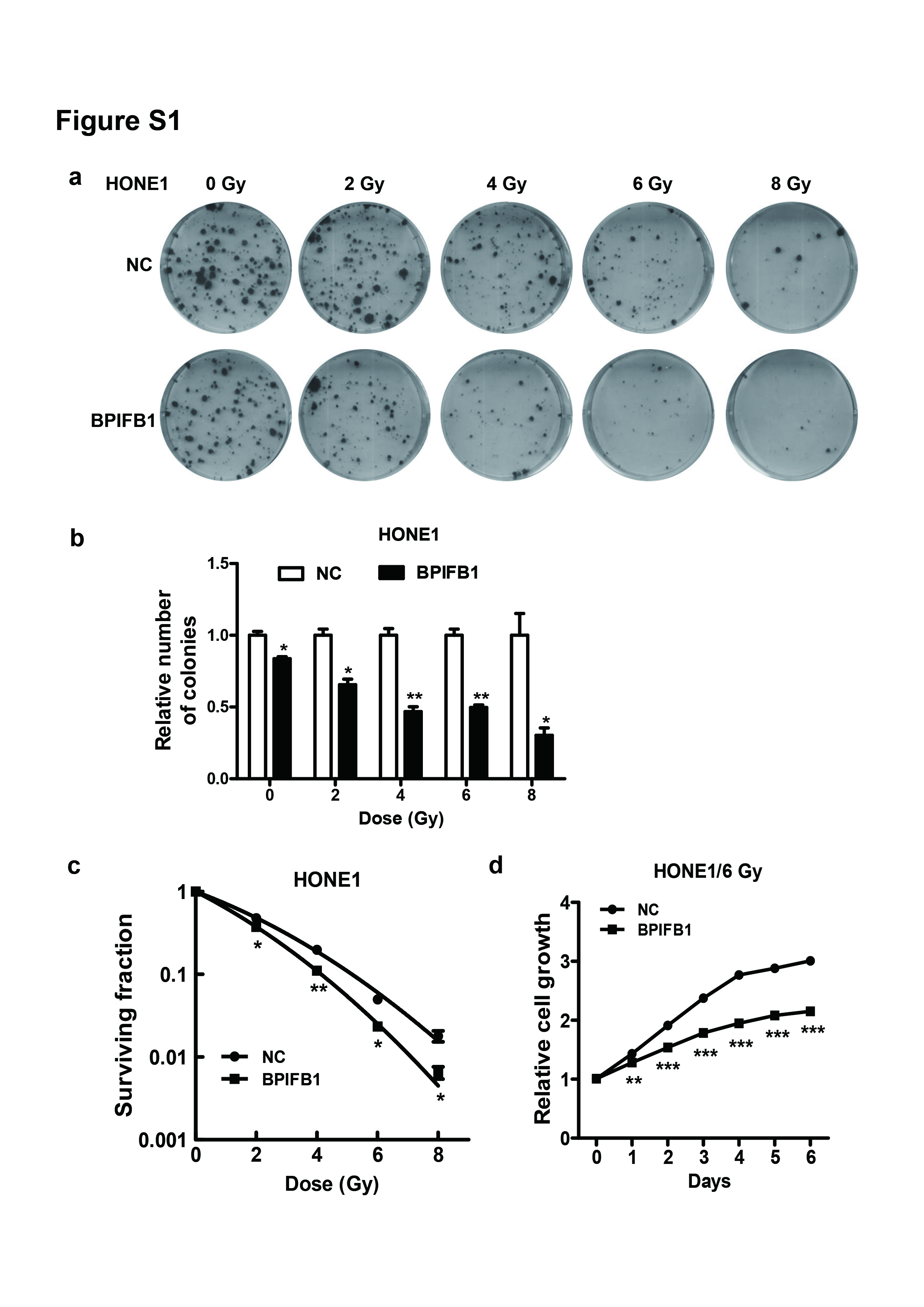

Supplement: Supplementary file 2 — supplementary Figure 1 [file 41419_2018_409_MOESM2_ESM.tif]

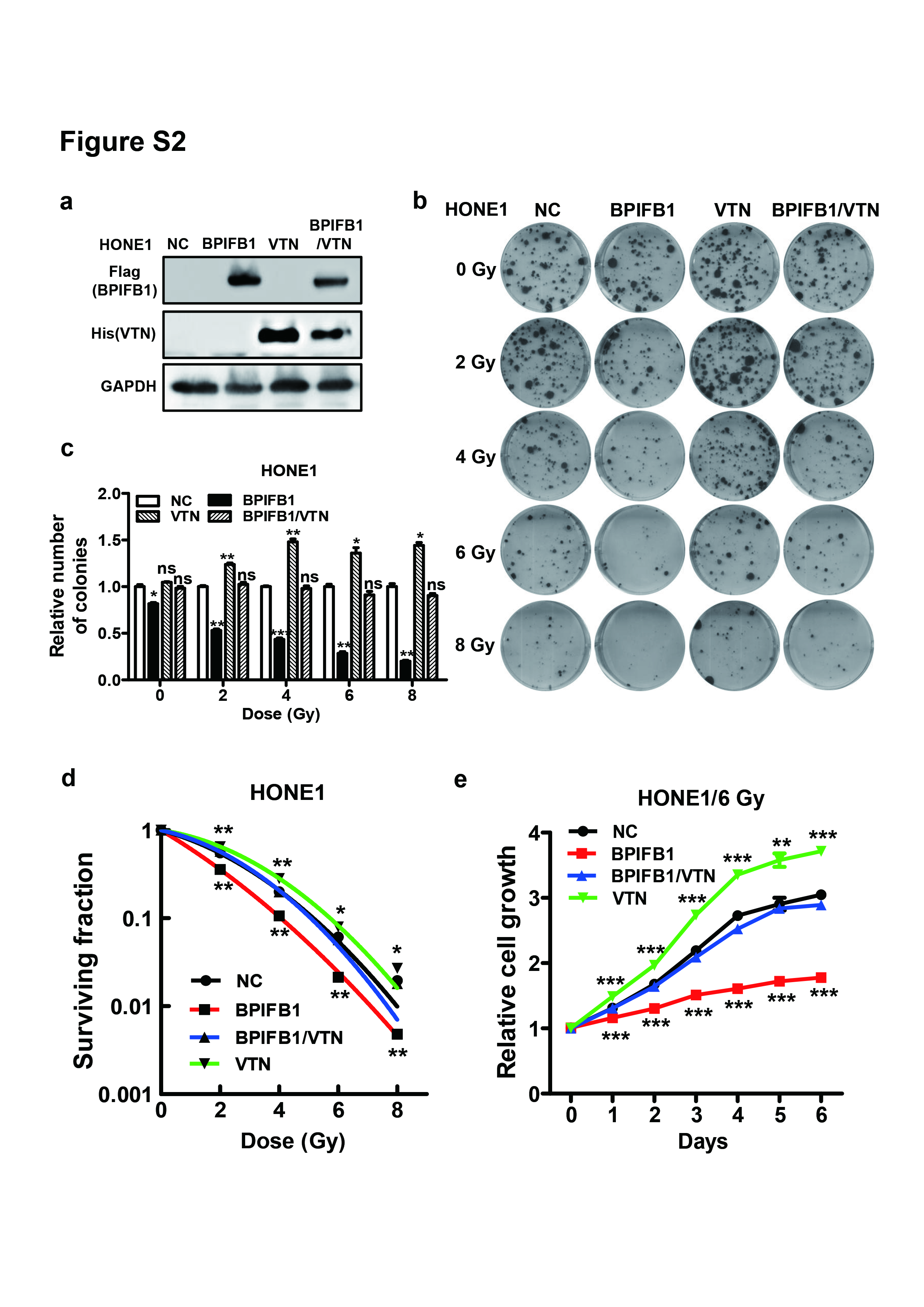

Supplement: Supplementary file 3 — supplementary Figure 2 [file 41419_2018_409_MOESM3_ESM.tif]

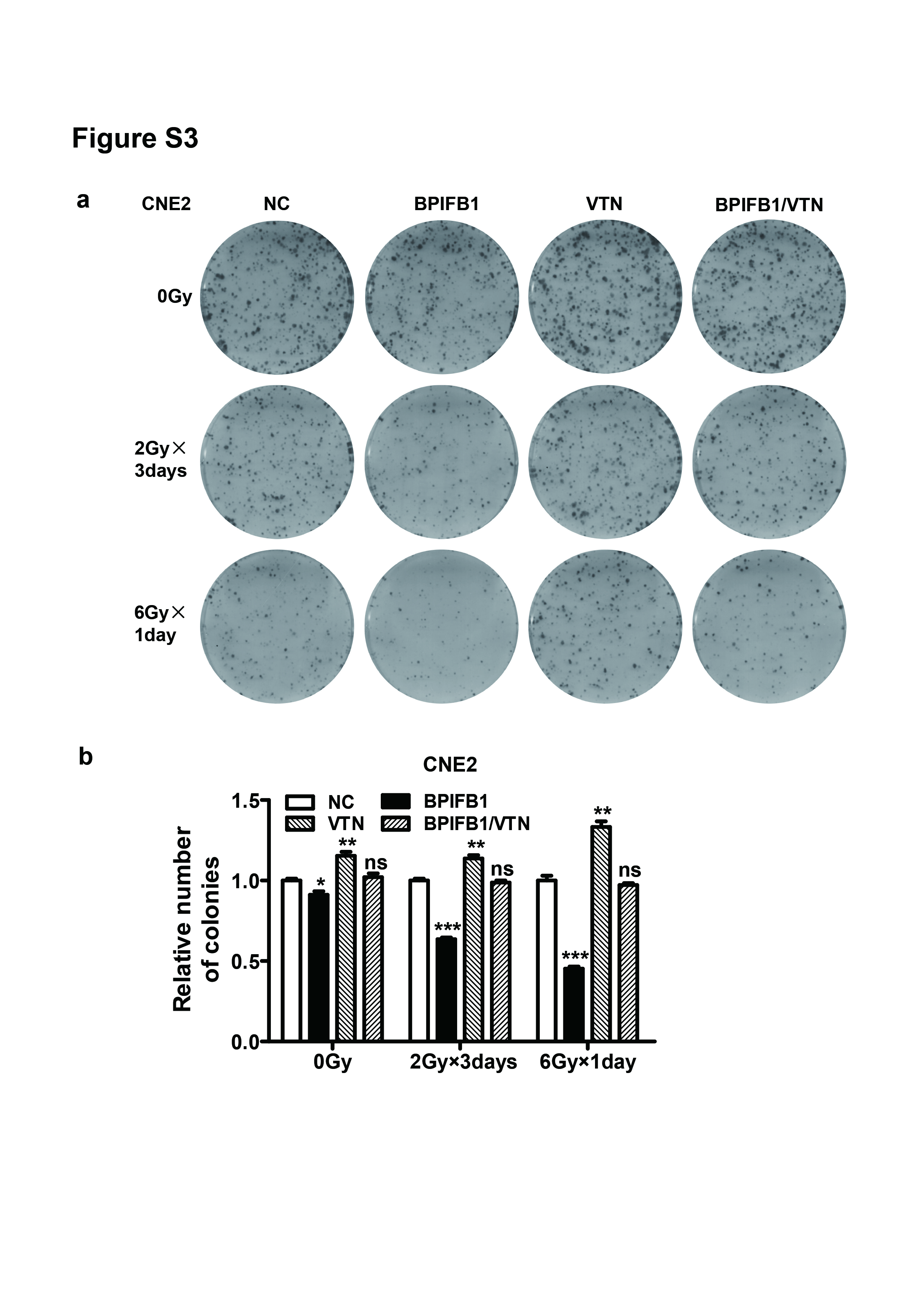

Supplement: Supplementary file 4 — supplementary Figure 3 [file 41419_2018_409_MOESM4_ESM.tif]

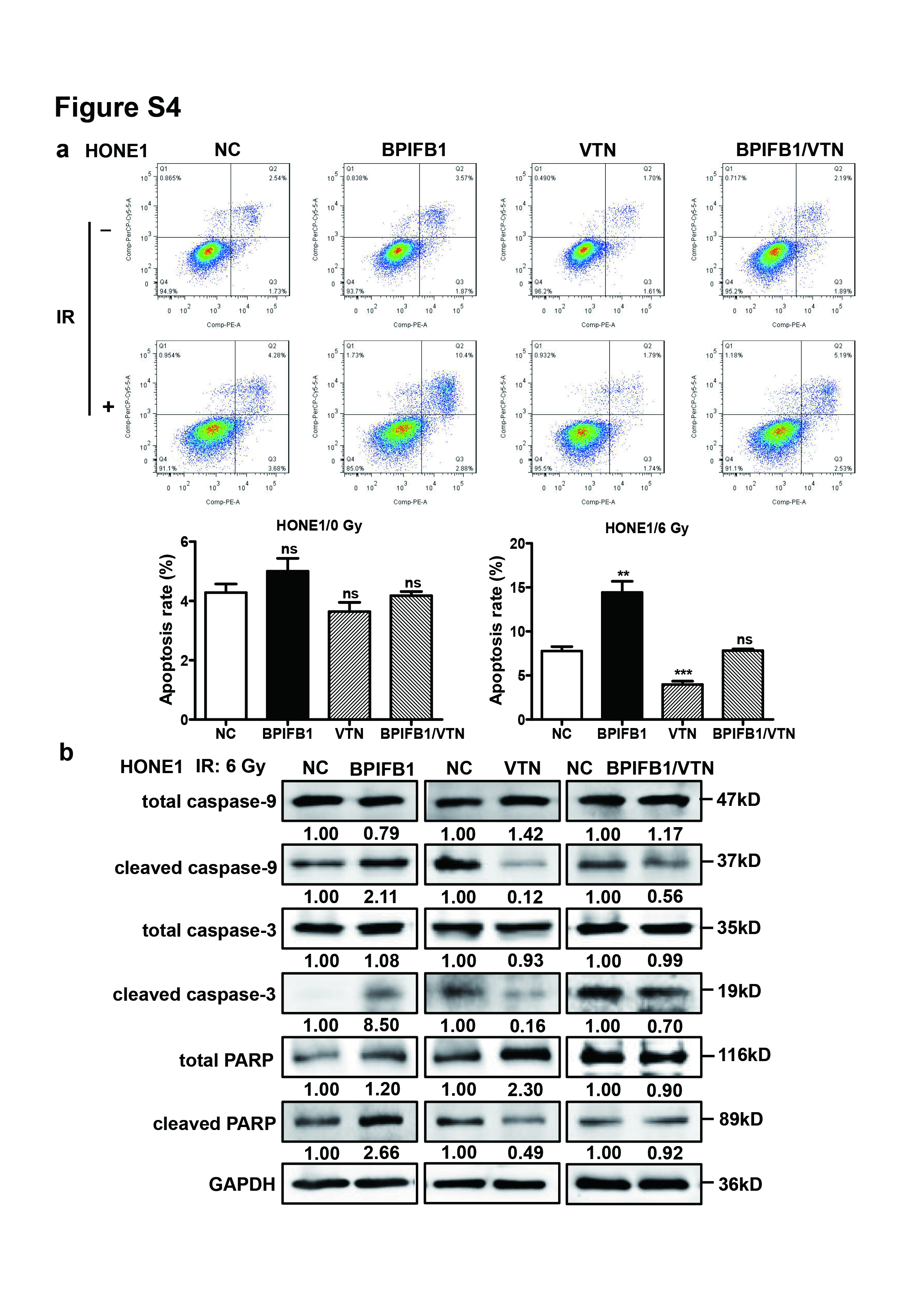

Supplement: Supplementary file 5 — supplementary Figure 4 [file 41419_2018_409_MOESM5_ESM.tif]

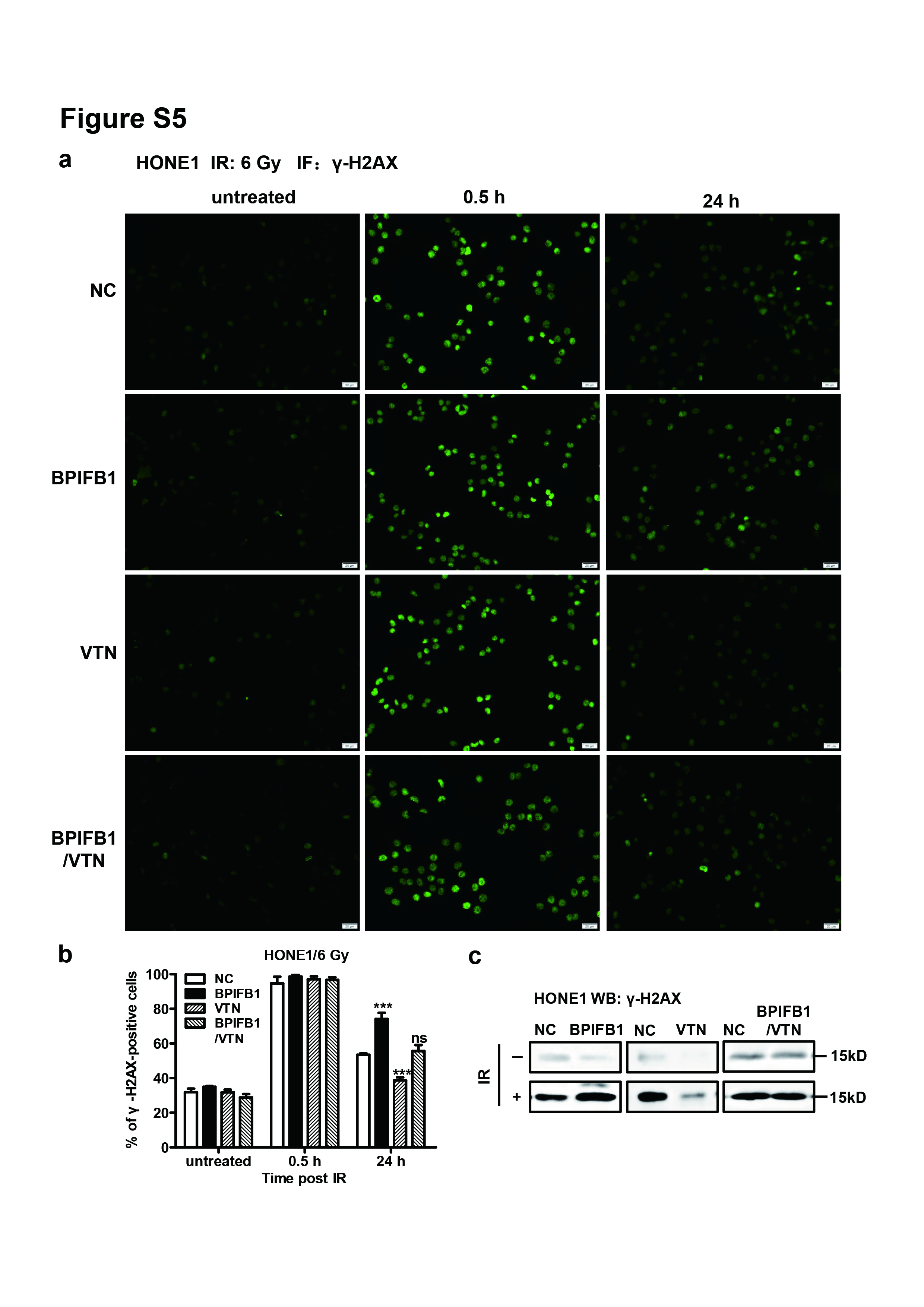

Supplement: Supplementary file 6 — supplementary Figure 5 [file 41419_2018_409_MOESM6_ESM.tif]

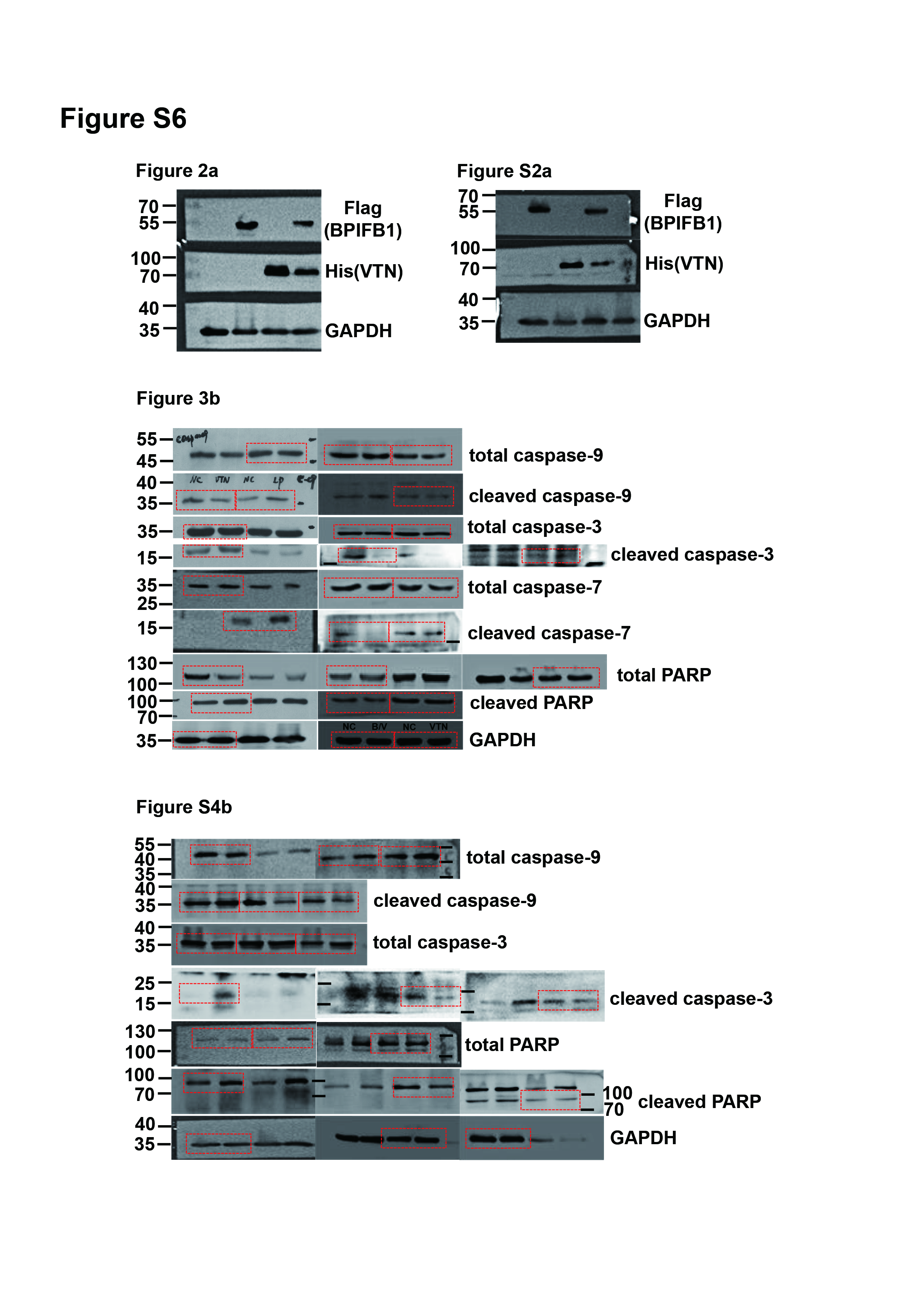

Supplement: Supplementary file 7 — supplementary Figure 6-1 [file 41419_2018_409_MOESM7_ESM.tif]

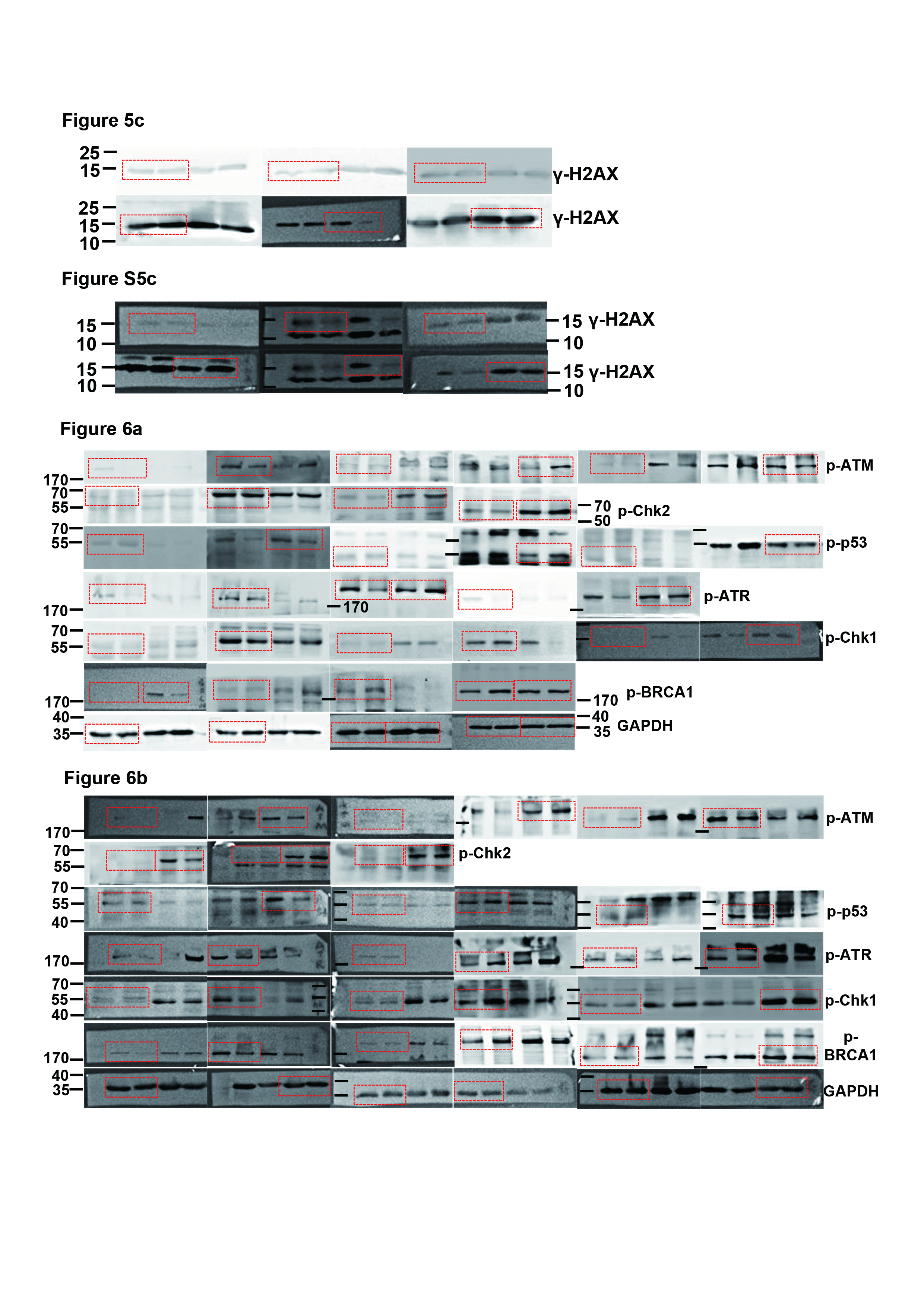

Supplement: Supplementary file 8 — supplementary Figure 6-2 [file 41419_2018_409_MOESM8_ESM.tif]
